# The Glasgow Microenvironment Score and risk and site of recurrence in TNM I–III colorectal cancer

**DOI:** 10.1038/s41416-022-02069-x

**Published:** 2022-12-07

**Authors:** P. G. Alexander, H. C. van Wyk, K. A. F. Pennel, J. Hay, D. C. McMillan, P. G. Horgan, C. S. D. Roxburgh, J. Edwards, J. H. Park

**Affiliations:** 1 School of Medicine, University of Glasgow, Glasgow, UK; 2 Institute of Cancer Sciences, University of Glasgow, Glasgow, UK; 3 Glasgow Tissue Research Facility, University of Glasgow, Queen Elizabeth University Hospital, Glasgow, UK

**Keywords:** Prognostic markers, Colon cancer, Rectal cancer

## Abstract

**Background:**

Glasgow Microenvironment Score (GMS) stratifies long-term survival into three groups based on tumour phenotype: peritumoural inflammation (Klintrup–Mäkinen (KM)) and tumour stroma percentage (TSP). However, it is not known if the location of disease recurrence is influenced by the GMS category.

**Methods:**

Seven hundred and eighty-three TNM I–III colorectal cancers (CRC) were included. GMS (GMS0—high KM; GMS1—low KM, low TSP; GMS2—low KM, high TSP) and cancer-specific survival (CSS), overall survival (OS) and disease recurrence were assessed using Cox regression analysis.

**Results:**

Of the 783 patients, 221 developed CRC recurrence; 65 developed local recurrence + systemic disease. GMS was independent for CSS (HR 1.50, 95% CI 1.17–1.92, *p* < 0.001) and OS (HR 1.23, 1.05–1.44, *p* = 0.01). Higher GMS category was associated with T-stage, N-stage, emergency presentation and venous invasion. GMS was independent for local+systemic recurrence (HR 11.53, 95% CI 1.45–91.85, *p* = 0.04) and distant-only recurrence (HR 3.01, 95% CI 1.59–5.71, *p* = 0.002). GMS 2 disease did not appear to have statistically better outcomes with adjuvant chemotherapy in high-risk disease.

**Conclusion:**

Although confounded by a higher rate of T4 and node-positive disease, GMS 1 and 2 are associated with an increased risk of local and distant recurrence. GMS is an independent poor prognostic indicator for recurrent colorectal cancer. Higher GMS patients may benefit from enhanced postoperative surveillance.

## Introduction

The disease burden posed by colorectal cancer (CRC) on healthcare worldwide is significant, with 1.8 million deaths attributed to the disease in 2018 [1]. The primary tool for guiding both prognosis and the multidisciplinary management of CRC is the TNM staging system, but this has its limitations and cannot account for wide variations in outcomes within each stage. Even with the addition of other commonly used clinicopathological features, such as venous invasion and common genetic markers, the prognostic ability remains poor [2].

In view of this need for further prognostic markers, the Glasgow Microenvironment Score (GMS) was developed, combining the beneficial prognostic marker of high peritumoural inflammation and the poor prognostic marker of high tumour stroma [3]. In terms of the consensus molecular subtypes (CMS) [4], high peritumoural inflammation is one of the defining features of the CMS1 (immune) subtype, whereas high tumour stroma represents the CMS4 (mesenchymal) subtype [5]. The poor prognosis of the CMS4 subgroup is due largely to its associated pro-angiogenic and immunosuppressive properties [6].

Recently, GMS has been validated in a large independent cohort and was found to be a prognostic indicator independent of TNM stage, venous invasion and measures of the systemic inflammatory response, with GMS 2 tumours shown to represent an additional high-risk feature in otherwise low-risk disease [7]. In addition, within a post hoc analysis of the SCOT Trial adjuvant chemotherapy study, the GMS aided in the selection of patients for adjuvant therapy; patients with GMS 0 appeared to derive greater benefit from FOLFOX compared to CAPOX, whereas patients with GMS 2 did not appear to obtain any benefit from either regimen [7]. Finally, a modified version of the GMS in colorectal cancer biopsy specimens has been shown to reflect that of the full resected specimen, indicating that it may be useful in aiding in the selection of patients for neoadjuvant therapy [8].

However, it is not yet known whether the GMS has the propensity to identify the likelihood of future recurrence or indicate potential sites of recurrence. There are data that suggest high stromal tumours, represented by GMS 2, have a higher rate of local recurrence [9, 10]. Given that prognosis is good in those with high peritumoural inflammation, represented by GMS 0, it is hypothesised that patients in this group would have a low recurrence rate in general. GMS 1 represents a heterogenous group with neither high peritumoural inflammation nor high TSP with an anticipated intermediate recurrence rate. Therefore, GMS may select patients who are more at risk of disease recurrence and who, as a result, may benefit from more intense postoperative surveillance.

Therefore, the aim of the present study was to examine the relationship between the GMS and patterns of recurrence in patients who have undergone resection of stage I–III colorectal cancer.

## Methods

The patients in this study were derived from a previously published cohort of 1000 patients who have undergone resection of colorectal adenocarcinoma between January 1997 and May 2013 in Glasgow Royal Infirmary [11]. The following exclusions were applied: mortality within 30 days, TNM 4 disease, and palliative or R1 resection (positive resection margins). Of the remaining 906 patients, pathology samples for GMS scoring were available for 783 tumours. The West of Scotland Research Ethics Committee provided ethical approval for the research.

### Study endpoints

The primary study endpoints were cancer-specific survival (CSS; measured from the date of surgery to date of death from cancer-specific cause or censor date), overall survival (OS; measured from the date of surgery to date of death from any cause or censor date) and colorectal cancer recurrence (recurrences were considered present on either radiological or pathological diagnosis, time from date of surgery to date of recurrence was calculated). Survival data were confirmed by review of electronic case notes and complete until 1 July 2020, which acted as the censor date. Data on the location of recurrence were collected from paper and electronic patient notes. Widespread recurrence was defined as more than one site of disease recurrence. As a secondary endpoint, the disease response of different GMS categories to adjuvant chemotherapy was compared with those not receiving chemotherapy.

### Clinicopathological data

Clinical characteristics and recurrence data were recorded from patient case notes, both paper and electronic, and the site of recurrence from imaging. Pathological data, including TNM stage and venous invasion (using elastic haematoxylin–eosin (H&E) staining, for which both intramural and extramural venous invasion were considered as present) were collected from pathology reports. As previously described [11], the modified Glasgow Prognostic Score was calculated using CRP (C-reactive Protein) and Albumin levels in whole venous blood obtained within the 30 days preceding surgery. Data were available regarding which patients received adjuvant chemotherapy, but not the regimen or duration of chemotherapy. The Petersen index was used to indicate low- and high-risk TNM stage II disease [12]: tumours with venous invasion or peritoneal involvement were assigned a score of 1, whereas tumour perforation was assigned a score of 2. Any individual with TNM III disease or TNM II with a Petersen index ≥2 was considered high-risk. The definition of emergency surgery was unplanned surgery on index hospital admission within 5 days.

### Scoring the GMS

Whole H&E-stained sections taken from the point of deepest invasion were scored manually for GMS using NDP view (Hamamatsu) after scanning slides onto a server using the Hamamatsu NanoZoomer at ×20 magnification (Welwyn Garden City, UK). GMS score was calculated according to Klintrup–Mäkinen grade (KM) and tumour stroma percentage (TSP), as described [13]. Briefly, KM was scored (by single investigator PGA, blinded to clinical data) semi-quantitatively at the tumour’s invasive margin as weak (no inflammatory cells present or mild increase only) or strong (presence of a band or cup-like infiltrate of inflammatory cells with evidence of tumour nest destruction). TSP was calculated by assigning the percentage area occupied, to the nearest 10%, by stroma vs tumour in the centre of the tumour at ×100 magnification, excluding areas of necrosis and mucin. This value was subsequently dichotomised into low stroma (≤50%) vs high stroma (>50%). The scores for KM and TSP were combined as follows: strong KM irrespective of TSP scored GMS 0; weak KM and low TSP scored GMS 1; weak KM and high TSP scored GMS 2.

Fifty cases were co-scored by a second investigator (HCvW), and for all scores, intra-class correlation co-efficient was >0.8.

### Mismatch repair (MMR) protein analysis

MMR status was assessed by immunohistochemistry according to UK NEQAS guidelines. Briefly, a tissue microarray comprised of four tumour-rich cores per patient was utilised, with immunohistochemical staining performed for MLH1, MSH2, MSH6 and PMS2 as previously described [14]. Intraepithelial immune cell staining was used as a positive control. Tumours were considered MMR proficient if there was strong nuclear staining with positive immune cells and were considered MMR deficient if there was loss of tumour nuclear staining while immune cells remained positive.

### Statistical analysis

All data were analysed using SPSS version 27.0 (IBM SPSS). Survival analysis was performed using Kaplan–Meier curves and log-rank analysis with adjustment for T-stage, N-stage and other clinicopathological features, where appropriate. Results are presented with hazard ratios (HRs) and 95% confidence intervals (CIs) calculated with univariate Cox regression analysis. Multivariate survival analysis was performed using a backward conditional stepwise model. A statistical significance threshold of *p* < 0.1 was used to identify variables for inclusion in the multivariate model. In-text results are given as HR, 95% CI for GMS 0 vs GMS 2, *p* value of log-rank analysis for the overall trend. Chi-squared analysis was performed to test associations between categorical variables and GMS. The study conformed to the REMARK guidelines [15] and the statistical significance value was set at *p* < 0.05.

## Results

Slides were available for scanning and subsequent GMS scoring for 783 tumours, out of a possible 906, with TNM I–III CRC. Compared with the missing slides, those with H&E slides available were more likely to have higher T-stage, more venous invasion and be colonic rather than rectal location (Supplementary Table S1). Of the available samples, 554 were colon cancers and 229 were rectal cancers. Clinicopathological characteristics for included patients are given in Table 1. Sixty-seven percent of patients were younger than 75 years at the time of surgery; 55% were male; 8% presented as an emergency and 61% were node negative. One hundred and thirty-two patients (17%) were GMS 0; 501 (64%) were GMS 1 and 149 (19%) were GMS 2. There were 477 deaths, of which 201 were related to CRC, and 221 developed recurrence. Of the recurrences, 66 patients developed local recurrence with or without systemic recurrence. An increasing GMS was associated with emergency presentation (*p* = 0.04), higher T- and N-stage (both *p* < 0.001), greater MMR deficiency (*p* = 0.02) and venous invasion (*p* < 0.001), (Table 1).

**Table 1:** Cancer-specific and overall survival in stage I–III colorectal cancer and associations of clinicopathological features with GMS (*N* = 783).

Clinicopathological characteristics		Cancer-specific survival				Overall survival				GMS category						
	*N* (%)^a^	Univariate HR (95% CI)	*p*	Multivariate HR (95% CI)	*p*	Univariate HR (95% CI)	*p*	Multivariate HR (95% CI)	*p*	0 (*n* = 132) *N* (%)^a^		1 (*n* = 501) *N* (%)		2 (*n* = 150) *N* (%)		Pearson *X*^2^
Age																
≤64	257 (33)									50	(38)	162	(32)	45	(30)	0.72
65–74	265 (34)									34	(26)	174	(35)	57	(38)	
≥75	261 (33)	1.39 (1.17–1.66)	**<0.001**	1.38 (1.14–1.67)	**<0.001**	1.85 (1.64–2.08)	**<0.001**	1.84 (1.62–2.09)	**<0.001**	48	(36)	165	(33)	48	(32)	
Gender																
Female	354 (45)									61	(46)	231	(46)	62	(41)	0.39
Male	429 (55)	1.16 (0.88–1.54)	0.29	–	–	1.12 (0.93–1.34)	0.23	–	–	71	(54)	270	(54)	88	(59)	
Presentation																
Elective	719 (92)									125	(95)	462	(92)	132	(88)	**0.04**
Emergency	64 (8)	2.11 (1.41–3.14)	**<0.001**	–	0.07	1.46 (1.09–1.96)	**0.012**	–	0.67	7	(5)	39	(8)	18	(12)	
TNM																
I	112 (14)									41	(31)	65	(13)	6	(4)	**<0.001**
II	368 (47)									62	(47)	249	(50)	57	(38)	
III	303 (39)	2.32 (1.83–2.93)	**<0.001**^b^	–	–	1.39 (1.21–1.59)	**<0.001**^b^	–	–	29	(22)	187	(37)	87	(58)	
T-stage																
T1	43 (6)									17	(13)	25	(5)	1	(1)	**<0.001**
T2	92 (12)									33	(25)	53	(11)	6	(4)	
T3	451 (58)									68	(52)	300	(60)	83	(55)	
T4	197 (25)	1.78 (1.44–2.19)	**<0.001**	1.31 (1.02–1.67)	**0.03**	1.33 (1.17–1.50)	**<0.001**	–	0.13	14	(11)	123	(25)	60	(40)	
N-stage																
N0	480 (61)									103	(78)	314	(63)	63	(42)	**<0.001**
N1	225 (29)									25	(19)	139	(28)	61	(41)	
N2	78 (10)	1.93 (1.61–2.31)	**<0.001**	1.89 (1.54–2.32)	<**0.001**	1.29 (1.13–1.46)	**<0.001**	1.37 (1.18–2.09)	<**0.001**	4	(3)	48	(10)	26	(17)	
Site																
Colon	554 (71)									93	(71)	359	(72)	102	(68)	0.63
Rectum	229 (29)	1.08 (0.80–1.45)	0.63	–	–	0.99 (0.81–1.20)	0.90	–	–	39	(30)	142	(28)	48	(32)	
Neoadjuvant therapy																
No	725 (93)									127	(96)	464	(93)	134	(91)	0.06
Yes	54 (7)	0.99 (0.59–1.68)	0.98	–	–	0.68 (0.45–1.03)	0.07	–	0.71	5	(4)	35	(7)	14	(10)	
Differentiation																
Well/mod	705 (91)									128	(97)	442	(89)	135	(90)	0.06
Poor	74 (9)	1.10 (0.68–1.79)	0.70	–	–	1.23 (0.91–1.66)	0.18	–	–	4	(3)	55	(11)	15	(10)	
MMR deficient																
No	550 (82)									92	(83)	363	(84)	95	(74)	**0.02**
Yes	121 (18)	1.41 (0.99–2.00)	0.05	–	0.08	1.35 (1.07–1.71)	**0.01**	1.30 (1.03–1.64)	**0.03**	19	(17)	68	(16)	34	(26)	
Venous invasion																
Absent	374 (48)									74	(56)	247	(49)	53	(35)	**<0.001**
Present	409 (52)	1.48 (1.12–1.97)	**0.006**	–	0.26	1.20 (1.00–1.44)	**0.047**	–	0.23	58	(44)	254	(51)	97	(65)	
mGPS																
0	500 (64)									88	(67)	323	(65)	89	(59)	0.19
1	160 (20)									22	(17)	108	(22)	30	(20)	
2	123 (16)	1.39 (1.17–1.66)	**<0.001**	1.22 (0.99–1.50)	0.06	1.44 (1.28–1.61)	**<0.001**	1.27 (1.12–1.44)	**<0.001**	22	(17)	70	(14)	31	(21)	
GMS																
0	132 (17)									–		–		–		–
1	501 (64)	1.95 (1.54–2.46)								–		–		–		
2	150 (19)		**<0.001**	1.54 (1.19–2.00)	**0.001**	1.41 (1.21–1.65)	<**0.001**	1.22 (1.07–1.49)	**0.006**	–		–		–		

^a^Percentages rounded to nearest whole number and may not total 100%.

^b^Not included in multivariate model as T-stage and N-stage are included separately.

Statistically significant *p* < 0.05 values are in bold.

**Fig. 1:**
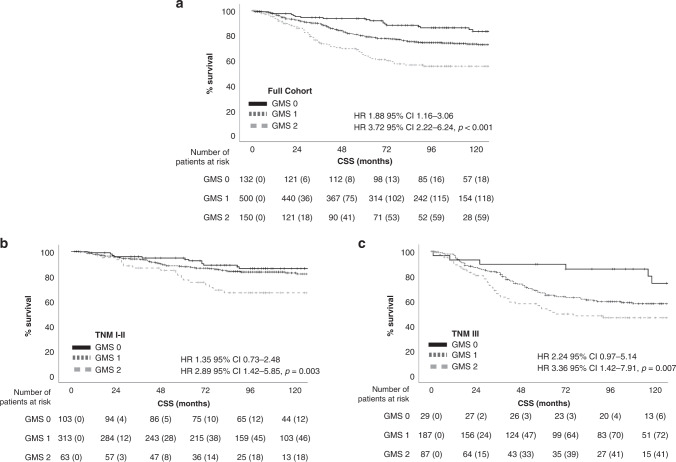
Cancer-specific survival according to GMS category. **a** Full cohort (*N* = 782); **b** TNM I-II (*N* = 479); **c** TNM III (*n* = 303).

Associations between GMS and CSS were assessed (Table 1 and Fig. 1a). GMS was able to stratify CSS in the whole cohort with 5-year CSS of 89% for GMS 0, 78% for GMS 1 and 61% for GMS 2 (GMS 0 vs GMS 2: HR 3.72 95% CI 2.22–6.24, *p* < 0.001). On multivariate analysis, GMS remained independent (*p* = 0.001) of age (*p* < 0.001), T-stage (*p* = 0.03), N-stage (*p* < 0.001) and mGPS (*p* = 0.06). Subgroup analysis was performed according to TNM stage, MMR status and primary tumour location (Table 2). GMS was able to stratify survival in early TNM I-II disease with 5-year CSS for GMS 0, 1 and 2 of 89%, 87% and 75%, respectively (GMS 0 vs GMS 2: HR 2.89 95% CI 1.42–5.85, *p* = 0.003, Fig. 1B); and TNM III disease with 5-year CSS for GMS 0, 1 and 2 of 90%, 63% and 50%, respectively (GMS 0 vs GMS 2: HR 3.36 95% CI 1.42–7.91, *p* = 0.006, Fig. 1C). GMS was also able to stratify MMR proficient and MMR deficient disease with 5-year CSS of 93%, 80% and 65%, and 95%, 75% and 59%, respectively (MMR proficient: GMS 0 vs GMS 2: HR 3.21 95% CI 1.76–5.84, *p* < 0.001; MMR deficient: GMS 0 vs GMS 2: HR 6.72 95% CI 1.53–29.58, *p* = 0.02). In addition, GMS was able to stratify CSS regardless of the use of adjuvant chemotherapy (No adjuvant therapy: GMS 0 vs GMS 2: HR 3.33 95% CI 1.91–5.82, *p* < 0.001; Adjuvant therapy: GMS 0 vs GMS 2: HR 11.54 95% CI 1.54–86.27, *p* = 0.02). It has been suggested that high stromal tumours respond poorly to standard chemotherapy, and it may be seen that the GMS 2 patients in the adjuvant chemotherapy group did not have a good outcome (Table 2). To further explore this, analysis was performed for high-risk patients for each GMS category according to whether the patients received adjuvant chemotherapy or not (Supplementary Table 2). The only group with a significant benefit from chemotherapy was GMS 1. Those with GMS 0 had a good outcome regardless of chemotherapy and those with GMS 2 did not have an improved outcome despite chemotherapy. Finally, GMS was able to stratify OS regardless of the site of the primary tumour (Colon cancer: GMS 0 vs GMS 2: HR 3.54 95% CI 1.92–6.51, *p* < 0.001; Rectal cancer: GMS 0 vs GMS 2: HR 4.17 95% CI 1.56–11.13, *p* = 0.004).

**Table 2:** Univariate survival for GMS according to TNM, MMR status, adjuvant chemotherapy and location of primary cancer (*N* = 783).

Group GMS category	*N*	Cancer-specific survival				Overall survival			
		5-year CSS (%; SE)	Events (*N* = 201)	HR (95% CI)	*p*	5-year OS (%; SE)	Events (*N* = 477)	HR (95% CI)	*p*
Full cohort				*Trend*	*<0.001*			*Trend*	*<0.001*
0	132	89 (3)	19	1.0 (reference)		75 (4)	67	1.0 (reference)	
1	501	78 (2)	122	1.88 (1.16–3.06)	**0.01**	63 (2)	310	1.40 (1.08-1.82)	**0.01**
2	149	61 (4)	60	3.72 (2.22–6.24)	**<0.001**	48 (4)	100	1.97 (1.44–2.69)	**<0.001**
TNM I–II				*Trend*	*0.003*			*Trend*	*0.09*
0	103	89 (3)	13	1.0 (reference)		74 (4)	51	1.0 (reference)	
1	314	87 (2)	50	1.35 (0.73–2.48)	0.34	69 (3)	183	1.26 (0.92–1.72)	0.15
2	63	75 (6)	19	2.89 (1.42–5.85)	**0.003**	58 (6)	38	1.59 (1.04–2.42)	**0.03**
TNM III				*Trend*	*0.007*			*Trend*	*0.006*
0	29	90 (6)	6	1.0 (reference)		79 (8)	16	1.0 (reference)	
1	187	63 (4)	72	2.24 (0.97–5.14)	0.06	54 (4)	127	1.67 (0.99–2.81)	0.05
2	86	50 (6)	41	3.36 (1.42–7.91)	**0.006**	41 (5)	62	2.32 (1.33–4.03)	**0.003**
MMR proficient				*Trend*	*<0.001*			*Trend*	*0.007*
0	92	93 (3)	15	1.0 (reference)		75 (5)	47	1.0 (reference)	
1	362	80 (2)	86	1.62 (0.94–2.81)	0.08	63 (3)	223	1.38 (1.01–1.89)	**0.045**
2	95	65 (5)	38	3.21 (1.76–5.84)	**<0.001**	49 (5)	62	1.84 (1.26–2.70)	**0.002**
MMR deficient				*Trend*	*0.024*			*Trend*	*0.02*
0	19	95 (5)	1	1.0 (reference)		68 (11)	12	1.0 (reference)	
1	68	75 (5)	34	3.81 (0.90–16.11)	0.07	60 (6)	50	1.21 (0.65–2.29)	0.55
2	34	59 (9)	19	6.72 (1.53–29.58)	**0.01**	38 (8)	28	2.23 (1.13–4.41)	**0.02**
No adjuvant chemo				*Trend*	*<0.001*			*Trend*	*0.001*
0	111	87 (3)	18	1.0 (reference)		70 (4)	63	1.0 (reference)	
1	375	78 (2)	88	1.59 (0.96–2.65)	0.07	61 (3)	252	1.33 (1.01–1.75)	**0.046**
2	99	63 (5)	40	3.33 (1.91–5.82)	**<0.001**	47 (5)	72	1.88 (1.34–2.64)	**<0.001**
Adjuvant chemo				*Trend*	*0.007*			*Trend*	*0.002*
0	21	100 (0)	1	1.0 (reference)		100 (0)	4	1.0 (reference)	
1	126	76 (4)	34	6.82 (0.93–49.86)	0.06	71 (4)	58	3.33 (1.21–9.20)	**0.02**
2	49	58 (7)	19	11.54 (1.54–86.27)	**0.02**	51 (7)	27	5.30 (1.84–15.26)	**0.002**
Colon cancer				*Trend*	*<0.001*			*Trend*	*0.002*
0	93	90 (3)	14	1.0 (reference)		77 (4)	47	1.0 (reference)	
1	359	79 (2)	85	1.78 (1.01–3.13)	**0.046**	64 (3)	225	1.40 (1.03–1.92)	**0.04**
2	102	61 (5)	40	3.54 (1.92–6.51)	**<0.001**	47 (5)	68	1.95 (1.34–2.83)	**<0.001**
Rectal cancer				*Trend*	*0.004*			*Trend*	*0.04*
0	39	89 (5)	5	1.0 (reference)		69 (7)	20	1.0 (reference)	
1	142	75 (4)	37	2.16 (0.85–5.50)	0.11	63 (4)	85	1.38 (0.84–2.24)	0.20
2	47	61 (8)	20	4.17 (1.56–11.13)	**0.004**	51 (7)	32	2.02 (1.15–3.54)	**0.015**

Statistically significant *p* < 0.05 values are in bold.

Next, associations between GMS and OS were assessed (Table 1). GMS was able to stratify OS in the whole cohort with 5-year OS of 75% for GMS 0, 63% for GMS 1 and 48% for GMS 2 (GMS 0 vs GMS 2: HR 1.97 95% CI 1.44–2.69, *p* < 0.001). On multivariate analysis, GMS was independent (*p* = 0.006) of age (*p* < 0.001), N-stage (*p* < 0.001), MMR deficiency (*p* = 0.03) and mGPS (*p* < 0.001). Subgroup analysis was performed according to TNM stage, MMR status, adjuvant therapy and primary tumour location (Table 2). GMS was able to stratify survival in TNM I–II disease with 5-year OS for GMS 0, 1 and 2 of 74, 69 and 58%, respectively (GMS 0 vs GMS 2: HR 1.59 95% CI 1.04–2.42, *p* = 0.03); and TNM III disease with 5-year OS for GMS 0, 1 and 2 of 79, 54 and 41%, respectively (GMS 0 vs GMS 2: HR 2.32 95% CI 1.33–4.03, *p* = 0.003). GMS was also able to stratify MMR-proficient and MMR-deficient disease with 5-year OS of 75, 63 and 49%, and 68, 60 and 38%, respectively (MMR proficient: GMS 0 vs GMS 2: HR 1.84 95% CI 1.26–2.70, *p* = 0.007; MMR deficient: GMS 0 vs GMS 2: HR 2.23 95% CI 1.13–4.41, *p* = 0.02). GMS was able to stratify OS regardless of the use of adjuvant chemotherapy (No adjuvant therapy: GMS 0 vs GMS 2: HR 1.88 95% CI 1.34–2.64, *p* < 0.001; Adjuvant therapy: GMS 0 vs GMS 2: HR 5.30 95% CI 1.84–15.26, *p* = 0.002) or the site of primary tumour (Colon cancer: GMS 0 vs GMS 2: HR 1.95 95% CI 1.34–2.83, *p* < 0.001; Rectal cancer: GMS 0 vs GMS 2: HR 2.02 95% CI 1.15–3.54, *p* = 0.015).

The relationship between pattern of recurrence and GMS was subsequently examined (Table 3). Overall, the recurrence rate for GMS 0 was 15% during the course of follow-up, compared with 27% in GMS 1 and 38% in GMS 2. The rates of local recurrence only GMS 0, 1 and 2 were 4, 5 and 7%, respectively, while for recurrence of local+systemic recurrences these were 1, 5 and 7%, respectively. Similarly, the rates for distant recurrence only were 10, 18 and 25%, respectively, for GMS 0, 1 and 2 (*p* < 0.001). In terms of specific recurrence location, GMS 0 had the highest recurrence-free rate of 85%, vs 73% for GMS 1 and 62% for GMS 2. The numbers were small for most individual locations, but the pattern was similar for liver, lung and widespread recurrences with the highest rates in GMS 2 and lowest in GMS 0.

**Table 3:** GMS and recurrence location (*n* = 737).

Group GMS category		GMS						
*Colon + Rectal cancer*	*N (%)*	0 (*n* = 125) *N* (%)^a^		1 (*n* = 474) *N* (%)		2 (*n* = 138) *N* (%)		Pearson *X*^2^
Recurrence								
None	540	106	(85)	348	(73)	86	(62)	
Local only	37	5	(4)	23	(5)	9	(7)	
Local + systemic	29	1	(1)	19	(4)	9	(7)	
Distant only	162	13	(10)	84	(18)	34	(25)	<0.001
Recurrence location								
None	540	106	(85)	348	(73)	86	(62)	–^b^
Local only	37	5	(4)	23	(5)	9	(7)	
Nodal	3	1	(1)	2	(1)	0	(0)	
Liver	57	3	(2)	39	(8)	15	(11)	
Lung	22	3	(2)	12	(3)	7	(5)	
Brain	6	1	(1)	5	(1)	0	(0)	
Widespread	74	6	(5)	46	(10)	22	(16)	
*Colon cancer only (n* *=* *544)*	*N*	0 (*n* = 89) *N* (%)^a^		1 (*n* = 338) *N* (%)		2 (*n* = 95) *N* (%)		
Recurrence								
None	384 (74)	73	(82)	251	(74)	60	(64)	
Local only	29	3	(3)	19	(6)	7	(7)	
Local + systemic	18	1	(1)	12	(4)	5	(5)	
Distant only	91 (17)	12	(14)	56	(17)	23	(24)	0.04
Recurrence location								
None	384 (74)	73	(86)	251	(82)	60	(72)	–^b^
Local only	29 (5)	3	(3)	18	(5)	7	(7)	
Nodal	3	1	(1)	2	(1)	0	(0)	
Liver	35 (6)	3	(3)	21	(6)	11	(12)	
Lung	12 (2)	2	(2)	7	(2)	3	(3)	
Brain	4	1	(1)	3	(1)	0	(0)	
Widespread	56	6	(7)	36	(11)	15	(15)	
*Rectal cancer only (n* = 229)	*N*	0 (*n* = 36) *N* (%)^a^		1 (*n* = 136) *N* (%)		2 (*n* = 43) *N* (%)		
Recurrence								
None	156 (73)	33	(92)	97	(71)	26	(61)	
Local only	8	2	(6)	4	(3)	2	(5)	
Local + systemic	11	0	(0)	7	(5)	4	(9)	
Distant only	40 (19)	1	(3)	28	(21)	11	(26)	<0.001
Recurrence location								
None	156 (68)	33	(92)	97	(71)	26	(61)	–^b^
Local only	7 (3)	2	(6)	4	(3)	1	(2)	
Nodal	0	–	–	–	–	–	–	
Liver	22 (10)	0	(0)	18	(13)	4	(9)	
Lung	10 (4)	1	(3)	5	(4)	4	(9)	
Brain	2	0	–	2	(2)	0	–	
Widespread	18	0	(0)	10	(7)	8	(19)	

^a^Total percentage may not equal 100 as it is rounded to the nearest whole number.

^b^No statistical analysis as cells with *n* < 6.

**Fig. 2:**
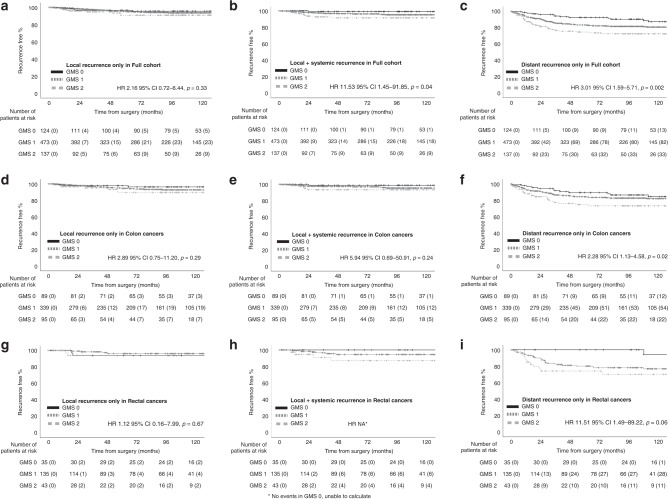
Recurrence risk in full cohort, colon cancers and rectal cancers, stratified by GMS. **a** Local recurrence only in full cohort; **b** local+systemic recurrence in full cohort; **c** distant recurrence only in full cohort; **d** local recurrence only in colon cancers; **e** local+systemic recurrence in colon cancers; **f** distant recurrence only in colon cancers; **g** local recurrence only in rectal cancers; **h** local+systemic recurrence in rectal cancers; **i** distant recurrence only in rectal cancers.

Cox regression analysis for recurrence risk was subsequently performed according to the location of recurrence in the full cohort (Table 4). On univariate analysis for local recurrence only, three variables were significant for recurrence risk and all remained independent on multivariate analysis: age (*p* = 0.01), T-stage (*p* = 0.02), and N-stage (*p* = 0.008). GMS was not significant for local recurrence only (Fig. 2a). GMS was, however, significant for local+systemic recurrence risk on multivariate analysis (*p* < 0.05, Fig. 2b), independent of T-stage (*p* = 0.009) and mGPS (*p* = 0.04). GMS was also significant in multivariate analysis for distant-only recurrence risk (*p* = 0.02, Fig. 2c), independent of N-stage (*p* < 0.001), venous invasion (*p* = 0.002) and mGPS (*p* = 0.02).

**Table 4:** Univariate and multivariate recurrence risk analysis in stage I–III colorectal cancer (full cohort).

Clinicopathological characteristics	Local recurrences only				Local + systemic				Distant only			
	Univariate HR (95% CI)	*p*	Multivariate HR (95% CI)	*p*	Univariate HR (95% CI)	*p*	Multivariate HR (95% CI)	*p*	Univariate HR (95% CI)	*p*	Multivariate HR (95% CI)	*p*
Age												
≤64												
65–74												
≥75	1.65 (1.09–2.50)	**0.02**	1.73 (1.14–2.64)	**0.01**	0.87 (0.55–1.38)	0.55	–	–	1.10 (0.88–1.36)	0.39	–	–
Gender												
Female												
Male	1.08 (0.56–2.07)	0.82	–	–	1.00 (0.48–2.09)	0.99	–	–	1.36 (0.95–1.94)	0.09	–	0.06
Presentation												
Elective												
Emergency	1.26 (0.39–4.11)	0.70	–	–	3.83 (1.55–9.46)	**0.004**	–	0.17	1.89 (1.12–3.19)	**0.02**	–	0.29
TNM												
I												
II (low risk)												
III (high risk)	2.20 (1.29–3.75)	**0.004**^a^	–	–	1.99 (1.10–3.60)	**0.02**^a^	–	–	2.01 (1.52–2.65)	**<0.001**^a^	–	–
T-stage												
T1												
T2												
T3												
T4	2.10 (1.27–3.46)	**0.004**	1.82 (1.09–3.06)	**0.02**	3.10 (1.66–5.77)	**<0.001**	2.39 (1.25–4.59)	**0.009**	1.50 (1.17–1.93)	**0.001**	–	0.39
N-stage												
N0												
N1												
N2	1.97 (1.29–2.99)	**0.02**	1.84 (1.18–2.89)	**0.008**	1.82 (1.13–2.94)	**0.01**	–	0.14	1.79 (1.43–2.25)	**<0.001**	1.58 (1.25–2.00)	<**0.001**
Differentiation												
Well/mod												
Poor	2.04 (0.85–4.90)	0.11	–	–	0.77 (0.18–3.23)	0.72	–	–	1.07 (0.59–1.93)	0.83	–	–
MMR status												
Proficient												
Deficient	1.71 (0.77–3.83)	0.19	–	–	0.93 (0.32–2.72)	0.90	–	–	1.19 (0.76–1.88)	0.45	–	–
Venous invasion												
Absent												
Present	0.81 (0.43–1.55)	0.53	–	–	2.14 (0.97–4.71)	0.06	–	0.35	2.07 (1.43–3.00)	**<0.001**	1.81 (1.23–2.65)	**0.002**
mGPS												
0												
1												
2	1.21 (0.79–1.85)	0.39	–	–	1.88 (1.22–2.90)	**0.004**	1.60 (1.01–2.52)	**0.04**	1.22 (0.98–1.53)	0.08	1.31 (1.04–1.64)	**0.02**
GMS												
0												
1												
2	1.49 (0.86–2.58)	0.15	–	–	2.52 (1.34–4.73)	**0.004**	1.90 (1.00–3.61)	**<0.05**	1.70 (1.27–2.28)	<**0.001**	1.41 (1.05–1.89)	**0.02**

^a^Not included in multivariate model as T-stage and N-stage are included separately.

Statistically significant *p* < 0.05 values are in bold.

Differences in recurrence patterns between colon and rectal cancers were subsequently assessed (Table 3 and Fig. 2). Comparing colon and rectal cancer recurrences, there was a higher number of liver metastases in rectal cancer than colon cancer (10 vs 7%, respectively), although this was not significant (*p* = 0.10). Furthermore, local recurrence rates, although small, were a similar number in both rectal and colon cancer (4 and 6%, respectively, *p* = 0.30) and when considering local with or without systemic recurrence, the rates were 9 and 9%, respectively. In colon cancers, the recurrence rate for GMS 0 was 18%, compared with 26% in GMS 1 and 36% in GMS 2. The rates of local recurrence only for GMS 0, 1 and 2 were 3, 6 and 7%, while those for local+systemic recurrence were 1, 4 and 5%, respectively. Similarly, the rates for distant recurrence only were 14, 17 and 24%, respectively, for GMS 0, 1 and 2 (*p* = 0.04). In terms of specific recurrence location, GMS 0 had the highest recurrence-free rate of 82%, vs 74% for GMS 1 and 64% for GMS 2. The numbers were small for most individual locations, but the pattern was similar for liver, lung and widespread recurrences with highest rates in GMS 2 and lowest in GMS 0. On univariate analysis, GMS was not significant for local recurrence only (*p* = 0.11, Fig. 2d and Table 5), nor for local+systemic recurrence (*p* = 0.07, Fig. 2e and Table 5). However, this was likely due to small numbers, since on analysis local recurrence with or without systemic recurrence, GMS was able to stratify recurrence risk, although the trend did not reach significance (HR 3.66 95% CI 1.18–11.36, *p* = 0.07, Supplementary Fig. 1). GMS was significant on univariate analysis for Distant recurrence only (*p* = 0.02, Fig. 2f and Table 5), although this was not independent of N-stage (*p* = 0.02), venous invasion (*p* = 0.03) or mGPS (*p* = 0.04).

**Table 5:** Univariate and multivariate recurrence risk analysis in stage I–III colon cancers (*N* = 554).

Clinicopathological characteristics	Local recurrences only				Local + systemic				Distant only			
	Univariate HR (95% CI)	*p*	Multivariate HR (95% CI)	*p*	Univariate HR (95% CI)	*p*	Multivariate HR (95% CI)^a^	*p*	Univariate HR (95% CI)	*p*	Multivariate HR (95% CI)	*p*
Age												
≤64												
65–74												
≥75	1.42 (0.89–2.25)	0.14	–	–	1.00 (0.57–1.78)	0.99	–	–	1.16 (0.90–1.50)	0.26	–	–
Gender												
Female												
Male	0.81 (0.39–1.67)	0.56	–	–	0.68 (0.27–1.73)	0.42	–	–	1.10 (0.72–1.66)	0.67	–	–
Presentation												
Elective												
Emergency	1.20 (0.36–3.95)	0.77	–	–	2.93 (0.96–8.91)	0.06	–	–	2.10 (1.22–3.61)	**0.007**	–	0.19
TNM												
I												
II (low risk)												
III (high risk)	2.14 (1.16–3.92)	**0.01**^b^	–	–	3.11 (1.33–7.24)	**0.009**^b^	–	–	1.90 (1.35–2.66)	**<0.001**^b^	–	–
T-stage												
T1												
T2												
T3												
T4	1.86 (1.06–3.26)	**0.03**	–	0.10	5.95 (2.24–15.82)	**<0.001**	–	–	1.71 (1.25–2.34)	**<0.001**	–	0.08
N-stage												
N0												
N1												
N2	1.92 (1.20–3.09)	**0.007**	1.71 (1.04–2.79)	**0.03**	2.28 (1.39–4.42)	**0.002**	–	–	1.61 (1.22–2.13)	**<0.001**	1.40 (1.05–1.89)	**0.02**
Differentiation												
Well/mod												
Poor	1.93 (0.73–5.05)	0.18	–	–	1.15 (0.27–5.01)	0.85	–	–	1.14 (0.59–2.19)	0.70	–	–
MMR status												
Proficient												
Deficient	1.54 (0.61–3.89)	0.36	–	–	0.92 (0.27–3.17)	0.89	–	–	1.07 (0.62–1.85)	0.80	–	–
Venous invasion												
Absent												
Present	0.82 (0.40–1.71)	0.61	–	–	3.48 (1.15–10.58)	**0.03**	–	–	2.04 (1.32–3.16)	**0.001**	1.65 (1.04–2.60)	**0.03**
mGPS												
0												
1												
2	1.17 (0.73–1.89)	0.52	–	–	1.81 (1.05–3.15)	**0.03**	–	–	1.39 (1.08–1.80)	**0.01**	1.32 (1.01–1.72)	**0.04**
GMS												
0												
1							–	–				
2	1.66 (0.89–3.08)	0.11	–	–	2.08 (0.94–4.57)	0.07			1.55 (1.09–2.19)	**0.02**	–	0.25

^a^Multivariate analysis not supported as only 18 events for Local + systemic.

^b^Not included in multivariate model as T-stage and N-stage are included separately.

Statistically significant *p* < 0.05 values are in bold.

In rectal cancers, the recurrence rate for GMS 0 was 8% during the course of follow-up, compared with 29% in GMS 1 and 39% in GMS 2. The rates of local recurrence only for GMS 0, 1 and 2 were 6, 3 and 5%, while those for local+systemic recurrence were 0, 7 and 9%, respectively. Similarly, the rates for distant recurrence only were 3, 21 and 26%, respectively, for GMS 0, 1 and 2 (*p* < 0.001). In terms of specific recurrence location, GMS 0 had the highest recurrence-free rate of 92%, vs 71% for GMS 1 and 61% for GMS 2. The numbers were small for most individual locations, but the pattern was similar for liver, lung and widespread recurrences with higher rates in GMS 2 and the lowest in GMS 0. On univariate analysis, GMS was not significant for local recurrence only (*p* = 0.92, Fig. 2g and Table 6). However, GMS was significant for local+systemic recurrence (*p* = 0.02, Fig. 2h and Table 6) and was the only variable to be significant for recurrence risk in this group other than mGPS. It was not possible to perform multivariate analysis for this group due to low event numbers (*N* = 11). For local recurrence with or without systemic recurrence, GMS trended towards significance, but was unable to stratify recurrence risk (HR 3.92 95% CI 0.78–19.77, *p* = 0.10, Supplementary Fig. 1). GMS was significant on multivariate analysis for distant recurrence only (HR 2.10, 1.23–3.56, *p* = 0.006, Fig. 2i and Table 6), independent of gender (*p* = 0.04) and N-stage (*p* < 0.001).

**Table 6:** Univariate and multivariate recurrence risk analysis in stage I–III rectal cancers (*N* = 229).

Clinicopathological characteristics	Local recurrences only				Local + systemic				Distant only			
	Univariate HR (95% CI)	*p*	Multivariate HR (95% CI)^b^	*p*	Univariate HR (95% CI)	*p*	Multivariate HR (95% CI)^b^	*p*	Univariate HR (95% CI)	*p*	Multivariate HR (95% CI)	*p*
Age												
≤64												
65–74												
≥75	2.76 (1.05–7.26)	**0.04**	–	–	0.68 (0.28–1.62)	0.38	–	–	0.99 (0.66–1.49)	0.97	–	–
Gender												
Female												
Male	5.00 (0.61–40.67)	0.13	–	–	2.13 (0.56–8.17)	0.27	–	–	2.44 (1.16–5.13)	**0.02**	2.21 (1.05–3.21)	**0.04**
Presentation												
Elective												
Emergency	–^a^	–	–	–	–^c^	–	–	–	–^c^	–	–	–
TNM												
I												
II (low risk)												
III (high risk)	2.59 (0.79–8.46)	0.12	–	–	1.16 (0.51–2.63)	0.73	–	–	2.23 (1.35–3.68)	**0.002**^d^	–	–
T-stage												
T1												
T2												
T3												
T4	3.13 (0.96–10.13)	0.06	–	–	1.72 (0.68–4.38)	0.25	–	–	1.23 (0.80–1.91)	0.35	–	–
N-stage												
N0												
N1												
N2	2.24 (0.90–5.59)	0.09	–	–	0.92 (0.37–2.27)	0.92	–	–	2.25 (1.50–3.37)	**<0.001**	2.10 (1.39–3.18)	<**0.001**
Differentiation												
Well/mod												
Poor	2.42 (0.30–19.69)	0.41	–	–	–^a^	–	–	–	0.87 (0.21–3.61)	0.85	–	–
MMR status												
Proficient												
Deficient	2.23 (0.43–11.50)	0.34	–	–	0.96 (0.12–7.94)	0.97	–	–	1.57 (0.68–3.63)	0.29	–	–
Venous invasion												
Absent												
Present	0.81 (0.20–3.25)	0.81	–	–	0.94 (0.29–3.10)	0.92	–	–	2.12 (1.06–4.24)	**0.03**	–	0.13
mGPS												
0												
1												
2	1.24 (0.45–3.37)	0.68	–	–	2.19 (1.09–4.39)	**0.03**	–	–	0.81 (0.46–1.43)	0.47	–	–
GMS												
0												
1												
2	1.06 (0.33–3.44)	0.92	–	–	3.50 (1.20–10.24)	**0.02**	–	–	2.10 (1.23–3.56)	**0.006**	1.99 (1.14–3.48)	**0.02**

^a^No events in GMS 0, not possible to perform Cox regression.

^b^Multivariate analysis not supported as only 8 events for Local only and 11 events for Local + systemic.

^c^Only three cases in the emergency group.

^d^Not included in multivariate model as T-stage and N-stage are included separately.

Statistically significant *p* < 0.05 values are in bold.

## Discussion

In this large, single-centre study, the GMS was observed to be an independent prognostic marker for TNM I-III CRC. An increasing GMS was associated with increased risk of recurrence overall. GMS was also able to identify those at risk of local recurrences with systemic involvement in the full cohort and in rectal cancers. The main advantage of GMS over other proposed scores is its ability to be performed on routine H&E-stained whole slides with no need for immunohistochemistry. Klintrup-Mäkinen grade has also been shown to strongly correlate with immunohistochemistry for other peritumoural immune cells (CD3 and CD8) [7, 16].

GMS 0, characterised by higher peritumoural inflammatory response, has been established as a prognostic marker conferring a survival benefit [17]. The same effect was observed in the current study with the lowest recurrence rate in this group. It must be noted, however, that the recurrence rate is not zero and whilst higher peritumoural inflammatory response is considered protective, there are clearly other factors at play in this group. Of note, the type of immune cells is not accounted for by this specific scoring system. Others have shown that polarisation of macrophages to M2 macrophages may be a poor prognostic sign [18]. Furthermore, these individuals that developed recurrence in spite of the beneficial phenotype of high KM may have had a more aggressive tumour biology. These, therefore, represent areas requiring further investigation and the combination of genetic profile and GMS is one of the planned future directions of study.

In the present study, patients with GMS 2 had the highest rates both of local and distant disease recurrence. Previous work suggests that this pathological phenotype, characterised by high TSP and accompanied by a poor immune response, denotes a mesenchymal subtype with poor prognosis and higher recurrence risk [5, 7, 9, 10, 19]. There are several confounding factors in this group with associations demonstrated between higher GMS and higher T-stage, N-stage and venous invasion, a finding also demonstrated in other studies [19]. GMS was not able to stratify risk of local only recurrence. However, GMS was an independent marker when comparing recurrence risk for both local+systemic recurrence and distant only recurrence. Furthermore, Kaplan–Meier curves for both CSS and OS display an early and sustained fall in survival in the GMS 2 group.

Given the high-risk nature of the GMS 2 phenotype, these tumours may warrant more aggressive follow-up with an enhanced surveillance programme, in order to detect recurrent disease at an earlier stage.

It was observed that 54 of the patients with rectal cancer also received neoadjuvant chemoradiotherapy and this may have had an impact on these recurrence figures [20]. Given the anatomy of colon and rectal cancers and the blood supply/venous drainage, it was hypothesised that local recurrences may be higher in the rectal cancer group whereas liver metastases may be higher in the colon cancer group [21]. Liver metastases were in fact proportionately higher in the rectal cancer group than the colon cancer group, although this was not significant. In addition, local recurrences were at a similar level in both groups.

Whilst not the primary aim of this study, the response of different GMS categories to chemotherapy was of interest, particularly in light of the findings in the SCOT trial [7]. In this cohort, the specific chemotherapy regimen was not known and the analysis therefore focused on chemotherapy vs no chemotherapy in high-risk colorectal cancer. GMS 1 was the only group found to benefit from chemotherapy. The numbers were relatively small in the GMS 0 subgroup. However, these patients were in a GMS category that have a naturally good prognosis according to phenotypic tumour assessment. The finding that the GMS 2 subgroup did not have a statistically better outcome following chemotherapy may indicate, as has been previously stated, that this group of tumours do not respond well to standard chemotherapy regimens and would benefit from novel strategies to combat high tumour stroma and the chemoresistance that this may offer [22].

In terms of limitations, this study was not performed in the context of the rigorous follow-up of a clinical trial and therefore, although data was taken from a prospectively maintained data set, it is possible that patients were lost to follow-up. Furthermore, a small number of patients received neoadjuvant therapy for rectal cancer (*n* = 54) and this is known to alter the appearance of the tumour microenvironment with the addition of fibrosis making assessment of TSP difficult [23]. However, only 14 of these were deemed to have high TSP. Additionally, GMS was independent of neoadjuvant therapy on multivariate analysis for survival. Finally, the chemotherapeutic regimen that patients received was not known, which limited further analysis of the adjuvant chemotherapy subgroup.

## Conclusion

GMS has been observed to associate with both local and systemic CRC recurrence. GMS was an independent prognostic indicator for disease recurrence at any location. The numbers for local disease recurrence were low. However, GMS was found to be an independent indicator of recurrence risk for both local+systemic recurrence and distant recurrence. Since GMS is a marker for recurrent colorectal cancer, patients with GMS 2 tumours may benefit from enhanced postoperative surveillance to aid the earlier detection of recurrent disease. Furthermore, patients with GMS 2 have not been found to respond well to standard chemotherapy; however, novel agents that may be of benefit remain to be investigated.

## Supplementary information

Reproducibility checklist

Supplementary Table S1

Supplementary Table S2

Supplementary Figure 1

REMARK checklist

## Data Availability

The datasets that formed the basis of this article are contained in the University of Glasgow’s MVLS Institute and are continually being updated with ongoing research. They contain patient-sensitive information and therefore cannot be made available on a public repository.
