# Supplementary material for: The Glasgow Microenvironment Score and risk and site of recurrence in TNM I–III colorectal cancer
Source: Br J Cancer. 2022 Dec 7;128(4):556–67. doi: 10.1038/s41416-022-02069-x (PMC9938140; doi:10.1038/s41416-022-02069-x)
Supplement: Supplementary file 2 — Supplementary Table S1 [file 41416_2022_2069_MOESM2_ESM.docx]

**Supplementary Table S1. Clinicopathological variables for patients with no H&E slides (*N*=123) vs those with H&E slides available for scanning (*N*=783).**

| Clinicopathological characteristics | | | |
| --- | --- | --- | --- |
|  | No H&E available | H&E available | Chi square |
|  | *N* (%)^a^ | *N* (%)^a^ | *P* |
| Age |  |  |  |
| ≤64 | 46 (37) | 257 (33) | 0.27 |
| 65-74 | 41 (33) | 265 (34) |  |
| ≥75 | 36 (29) | 261 (33) |  |
| Gender |  |  |  |
| Female | 59 (48) | 354 (45) | 0.57 |
| Male | 64 (52) | 429 (55) |  |
| Presentation |  |  |  |
| Elective | 113 (92) | 719 (92) | 0.99 |
| Emergency | 10 (8) | 64 (8) |  |
| TNM |  |  |  |
| I  II (low risk) | 34 (28)  48 (39) | 112 (14)  368 (47) | **0.006** |
| III (high risk) | 41 (33) | 303 (39) |  |
| T-stage |  |  |  |
| T1 | 22 (18) | 43 (6) | **<0.001** |
| T2 | 17 (12) | 92 (12) |  |
| T3 | 65 (53) | 451 (58) |  |
| T4 | 19 (15) | 197 (25) |  |
| N-stage |  |  |  |
| N0 | 82 (67) | 480 (61) | 0.47 |
| N1 | 28 (23) | 225 (29) |  |
| N2 | 13 (11) | 78 (10) |  |
| Site |  |  |  |
| Colon | 68 (55) | 554 (71) | **0.001** |
| Rectum | 55 (45) | 229 (29) |  |
| Differentiation |  |  |  |
| Well/mod | 112 (93) | 705 (91) | 0.47 |
| Poor | 9 (7) | 74 (9) |  |
| Venous invasion |  |  |  |
| Absent | 71 (58) | 374 (48) | **0.04** |
| Present | 52 (42) | 409 (52) |  |
| mGPS |  |  |  |
| 0 | 83 (67) | 500 (64) | 0.45 |
| 1 | 23 (19) | 160 (20) |  |
| 2 | 17 (14) | 123 (16) |  |
| GMS  0  1  2 |  |  |  |
|  | -  -  - | 132 (17)  501 (64)  150 (19) | - |
|  |  |  |  |

^a^percentages rounded to nearest whole number and may not total 100%
