# Supplementary material for: The Glasgow Microenvironment Score and risk and site of recurrence in TNM I–III colorectal cancer
Source: Br J Cancer. 2022 Dec 7;128(4):556–67. doi: 10.1038/s41416-022-02069-x (PMC9938140; doi:10.1038/s41416-022-02069-x)
Supplement: Supplementary file 3 — Supplementary Table S2 [file 41416_2022_2069_MOESM3_ESM.docx]

**Supplementary Table S2. Univariate CSS survival for adjuvant chemotherapy vs no chemotherapy in high risk TNM according to GMS.**

| Group |  | Cancer-specific Survival | | | | |
| --- | --- | --- | --- | --- | --- | --- |
|  | *N* | 10-year CSS  (%; SE) | Events | HR (95% CI) | *P* |  |
| GMS 0 |  |  |  |  |  |  |
| No adjuvant chemotherapy | 17 | 62 (14) | 5 | 1.0 (reference) |  |  |
| Adjuvant chemotherapy | 17 | 92 (8) | 1 | 0.14 (0.16-1.21) | 0.07 |  |
|  |  |  |  |  |  |  |
| GMS 1 |  |  |  |  |  |  |
| No adjuvant chemotherapy | 119 | 51 (5) | 48 | 1.0 (reference) |  |  |
| Adjuvant chemotherapy | 104 | 66 (5) | 34 | 0.63 (0.40-0.97) | **0.04** |  |
|  |  |  |  |  |  |  |
| GMS 2 |  |  |  |  |  |  |
| No adjuvant chemotherapy | 51 | 39 (8) | 27 | 1.0 (reference) |  |  |
| Adjuvant chemotherapy | 44 | 59 (8) | 17 | 0.57 (0.31-1.04) | 0.07 |  |
|  |  |  |  |  |  |  |
