# Supplementary material for: The Glasgow Microenvironment Score and risk and site of recurrence in TNM I–III colorectal cancer
Source: Br J Cancer. 2022 Dec 7;128(4):556–67. doi: 10.1038/s41416-022-02069-x (PMC9938140; doi:10.1038/s41416-022-02069-x)
Supplement: Supplementary file 4 — Supplementary Figure 1 [file 41416_2022_2069_MOESM4_ESM.pptx]

## Slide 1
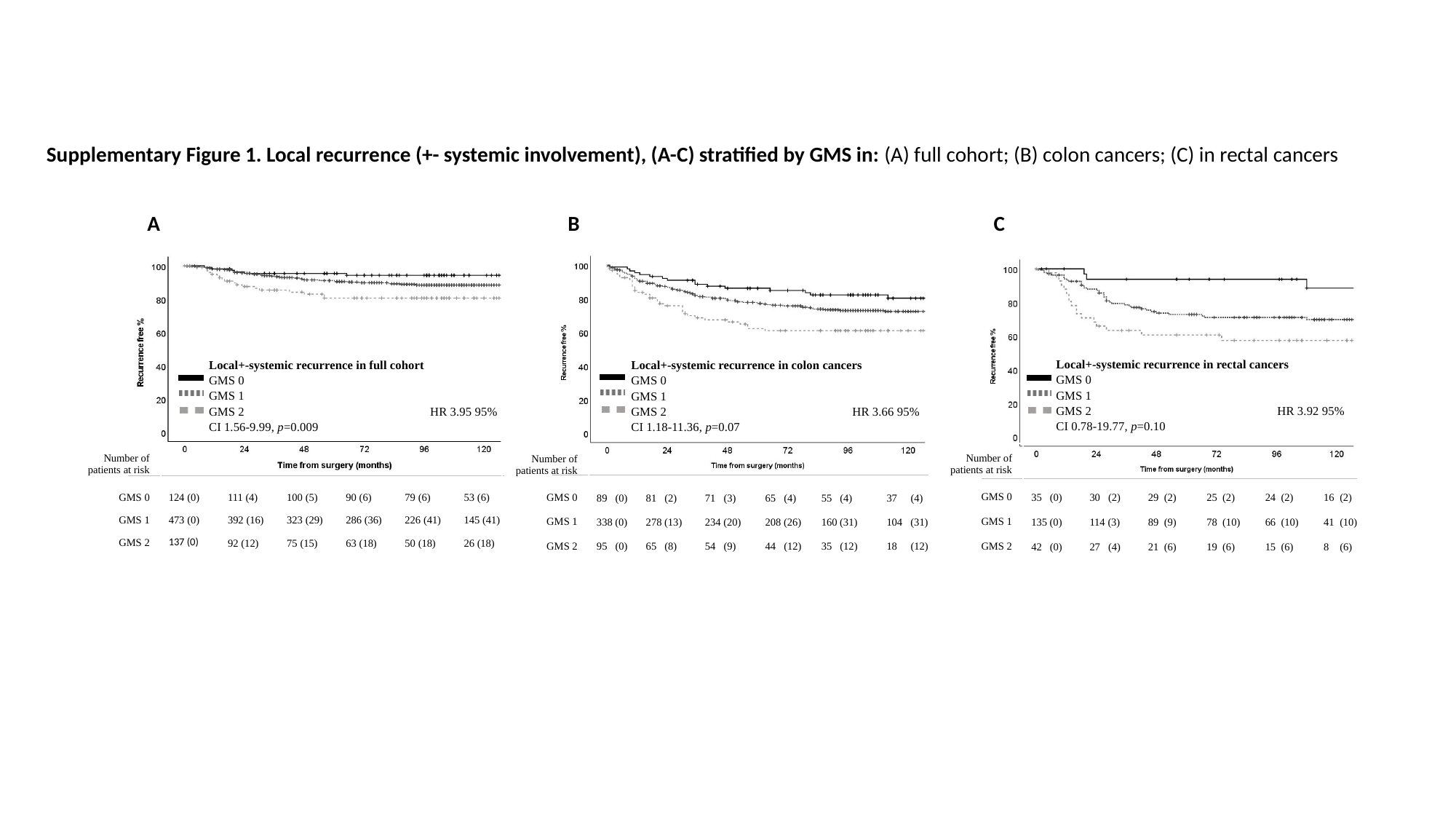

Supplementary Figure 1. Local recurrence (+- systemic involvement), (A-C) stratified by GMS in: (A) full cohort; (B) colon cancers; (C) in rectal cancers
A
B
C
Local+-systemic recurrence in rectal cancers
GMS 0
GMS 1
GMS 2		 HR 3.92 95% CI 0.78-19.77, p=0.10
Local+-systemic recurrence in full cohort
GMS 0
GMS 1
GMS 2		 HR 3.95 95% CI 1.56-9.99, p=0.009
Local+-systemic recurrence in colon cancers
GMS 0
GMS 1
GMS 2		 HR 3.66 95% CI 1.18-11.36, p=0.07
| Number of patients at risk | | | | | | |
| --- | --- | --- | --- | --- | --- | --- |
| GMS 0 | 124 (0) | 111 (4) | 100 (5) | 90 (6) | 79 (6) | 53 (6) |
| GMS 1 | 473 (0) | 392 (16) | 323 (29) | 286 (36) | 226 (41) | 145 (41) |
| GMS 2 | 137 (0) | 92 (12) | 75 (15) | 63 (18) | 50 (18) | 26 (18) |
| Number of patients at risk | | | | | | |
| --- | --- | --- | --- | --- | --- | --- |
| GMS 0 | 35 (0) | 30 (2) | 29 (2) | 25 (2) | 24 (2) | 16 (2) |
| GMS 1 | 135 (0) | 114 (3) | 89 (9) | 78 (10) | 66 (10) | 41 (10) |
| GMS 2 | 42 (0) | 27 (4) | 21 (6) | 19 (6) | 15 (6) | 8 (6) |
| Number of patients at risk | | | | | | |
| --- | --- | --- | --- | --- | --- | --- |
| GMS 0 | 89 (0) | 81 (2) | 71 (3) | 65 (4) | 55 (4) | 37 (4) |
| GMS 1 | 338 (0) | 278 (13) | 234 (20) | 208 (26) | 160 (31) | 104 (31) |
| GMS 2 | 95 (0) | 65 (8) | 54 (9) | 44 (12) | 35 (12) | 18 (12) |
